# Development of a Secondary Prevention Smartphone App for Students With Unhealthy Alcohol Use: Results From a Qualitative Assessment

**DOI:** 10.2196/41088

**Published:** 2023-03-07

**Authors:** Nicolas Bertholet, Elodie Schmutz, John A Cunningham, Jennifer McNeely, Gerhard Gmel, Jean-Bernard Daeppen, Véronique S Grazioli

**Affiliations:** 1 Addiction Medicine Unit Department of Psychiatry Lausanne University Hospital Lausanne Switzerland; 2 Faculty of Biology and Medicine University of Lausanne Lausanne Switzerland; 3 National Addiction Centre Institute of Psychiatry, Psychology and Neuroscience Kings College London London United Kingdom; 4 Centre for Addiction and Mental Health Toronto, ON Canada; 5 Section on Tobacco, Alcohol and Drug Use Department of Population Health New York University Grossman School of Medicine New York City, NY United States; 6 Department of Vulnerabilities and Social Medicine Center for Primary Care and Public Health University of Lausanne Lausanne Switzerland

**Keywords:** app, alcohol-related secondary prevention, university students, tertiary students, qualitative, alcohol, mHealth, mobile app, smartphone, mobile phone

## Abstract

**Background:**

Despite considerable efforts devoted to the development of prevention interventions aiming at reducing unhealthy alcohol use in tertiary students, their delivery remains often challenging. Interventions including information technology are promising given their potential to reach large parts of the population.

**Objective:**

This study aims to develop a secondary prevention smartphone app with an iterative qualitative design involving the target population.

**Methods:**

The app development process included testing a first prototype and a second prototype, developed based on the results of 2 consecutive qualitative assessments. Participants (aged ≥18 years, screened positive for unhealthy alcohol use) were students from 4 tertiary education institutions in the French-speaking part of Switzerland. Participants tested prototype 1 or prototype 2 or both and provided feedback in 1-to-1 semistructured interviews after 2-3 weeks of testing.

**Results:**

The mean age of the participants was 23.3 years. A total of 9 students (4/9 female) tested prototype 1 and participated in qualitative interviews. A total of 11 students (6/11 female) tested prototype 2 (6 who tested prototype 1 and 5 new) and participated in semistructured interviews. Content analysis identified 6 main themes: “General Acceptance of the App,” “Importance of the Targeted and Relevant App Content,” “Importance of Credibility,” “Importance of the App Usability,” “Importance of a Simple and Attractive Design,” “Importance of Notifications to Ensure App Use over Time.” Besides a general acceptance of the app, these themes reflected participants’ recommendations toward increased usability; to improve the design; to include useful and rewarding contents; to make the app look serious and credible; and to add notifications to ensure its use over time. A total of 11 students tested prototype 2 (6 who tested prototype 1 and 5 new) and participated in semistructured interviews. The 6 same themes emerged from the analysis. Participants from phase 1 generally found the design and content of the app improved.

**Conclusions:**

Students recommend prevention smartphone apps to be easy to use, useful, rewarding, serious, and credible. These findings may be important to consider when developing prevention smartphone apps to increase the likelihood of app use over time.

**Trial Registration:**

ISRCTN registry 10007691; https://www.isrctn.com/ISRCTN10007691

**International Registered Report Identifier (IRRID):**

RR2-10.1186/s13063-020-4145-2

## Introduction

Unhealthy alcohol use is a leading cause of morbidity and mortality among young people, including among students in whom unhealthy alcohol use is associated with academic impairment, damage to self and others (assaults, unprotected sex, suicide, interpersonal violence), and institutional costs (property damage) [1]. Despite prevention efforts, consequences tend to increase over time [2]. In Switzerland, a significant proportion of the mortality among young people is attributed to alcohol [3,4].

The screening and brief intervention has demonstrated efficacy in primary care as an approach for nontreatment-seeking individuals [5-7]. Information technology has the potential to offer access to the screening and brief intervention to larger parts of the general population [8,9].

According to a 2019 Pew Research Center report [10], 76% of people in advanced economies are reporting smartphone ownership. The proportion of the population owning a smartphone is especially high for 18-34-year olds: 95% in the United States, 90% in Canada, 97% in France, 98% in Germany, 98% in Italy, 93% in the United Kingdom, 95% in Spain, and 99% in the Netherlands [10]. Given its widespread use, the smartphone may be an excellent tool to disseminate interventions, especially among young individuals. In a context in which there is a demand for electronic interventions [11], the development of smartphones offers an opportunity for more proactive interventions, with the potential for multiple contact at the user’s convenience, which may help increase the intensity of interventions.

Although the development of smartphone apps related to alcohol use has exploded, there is limited evidence regarding their efficacy to reduce unhealthy alcohol use [12,13]. The scarcity of evidence is particularly noticeable at a time when numerous apps are being developed and released. In addition, among the current apps focusing on alcohol reduction, few contain evidence-based behavior change techniques [14] and even thoughtfully developed apps can be associated with unanticipated adverse effects [15].

We are conducting a larger mixed methods study aiming to develop and test a smartphone app for unhealthy alcohol use among tertiary students through a randomized trial [16]. This paper presents the development of the smartphone app targeting unhealthy alcohol use. In this qualitative study, students were involved in an iterative process aiming at developing the app suitable to its target population. The developed app is currently being tested in a randomized trial.

## Methods

### Overview

We developed and tested in a previous pilot study a smartphone app targeting unhealthy alcohol use, based on a web-based intervention with demonstrated efficacy among young individuals [17,18]. We further developed this existing proactive secondary prevention smartphone app, taking into account the limitations observed during the pilot studies. The app was designed to offer additional features, taking advantage of the specificities of smartphones (ie, increased level of personalization and immediacy or access to intervention material in situations outside of the reach of face-to-face or computer interventions). As for numerous electronic interventions targeting unhealthy alcohol use, this app includes a social norms intervention [1,19-21], a type of intervention considered effective for college students [22]. The social norms intervention consists of normative feedback. The user’s alcohol consumption is compared with the alcohol consumption of people of the same age and sex in Switzerland, based on Swiss population data [23]. Normative feedback is provided for the volume of drinking (number of drinks per week) and for the frequency of heavy drinking episodes (frequency of episodes with ≥5 drinks [men]/≥4 drinks [women]). The app also provides personalized feedback on the risk of harm. Personalized feedback is considered one of the possible mechanisms of brief interventions to reduce alcohol use [24,25]. In addition, the app was designed to encourage self-efficacy through autonomous goal setting. It also provides additional information and resources to users willing or needing more: information and contact options for local addiction and mental health treatment resources, including student health centers, are listed.

**Figure 1:**
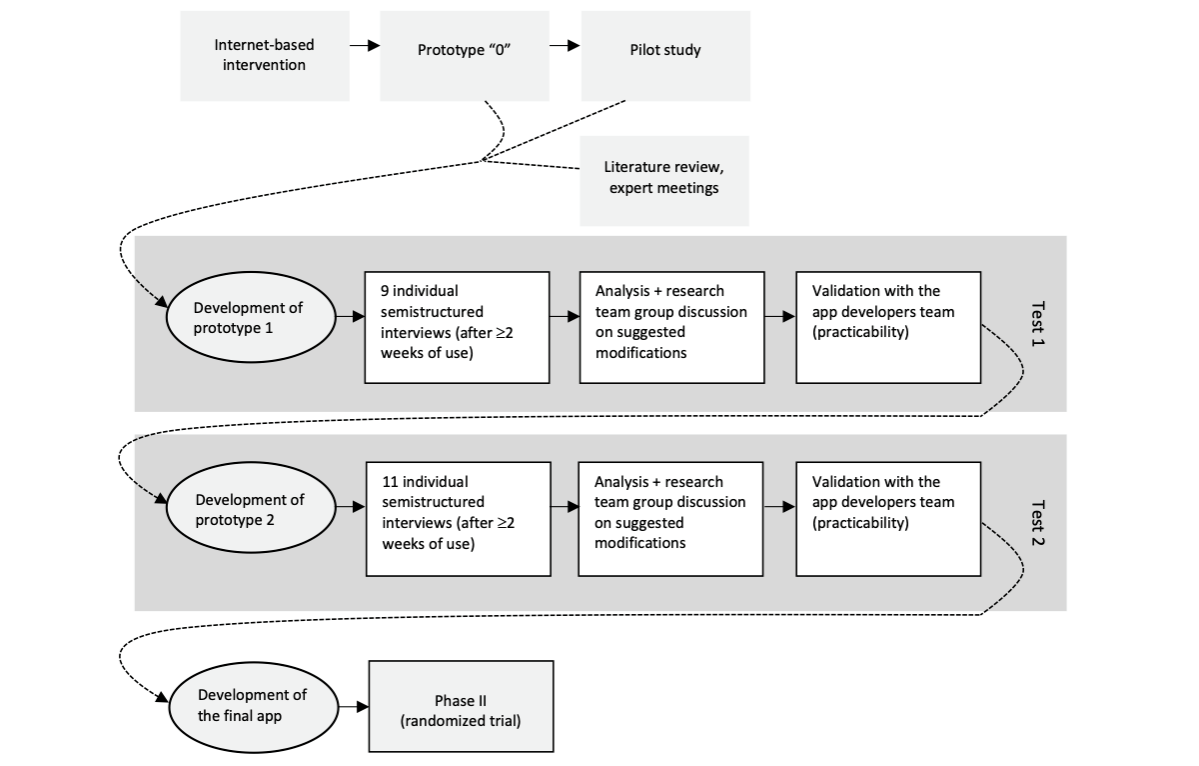
The app development iterative process.

The development of the app included the following iterative steps: (1) development of the initial prototype based on the app tested in pilot studies [26,27]; (2) test of the initial prototype in the target population and qualitative assessment; (3) app adjustments based on qualitative findings; (4) test of the second version (prototype 2) of the app in the target population and qualitative assessment; (5) final adjustments based on qualitative findings. Figure 1 shows the app development process.

The development of the initial prototype (ie, prototype 1 in Figure1) was conducted through regular meetings between members of the research team and developers. Throughout the development process, informal testing was performed by members of the research team, developers, colleagues, and students working in our unit to ensure usability as well as text and design appropriateness. Notably, the aim was to make the app more “active” and to send messages to participants (ie, notifications) following prespecified scenarios, knowing that students appear acceptant of receiving such messages [28] and that smartphone app users identify prompts as important [29]. Two groups of students, member of the app’s target population, were recruited to test the app in 1 (prototype 1) or 2 (prototype 2) of 2 tests and participate in in-depth interviews about the content of the app.

The app development started in October 2018 and a first version was ready for testing in February 2019. The final version was ready in November 2020 (due to the COVID-19 pandemic, the randomized trial was delayed and started in May 2021).

### Materials

The initial version of the app content in prototype 1 comprised 5 modules: (1) personalized feedback on self-reported alcohol consumption, with normative feedback, feedback on the calorific content of the reported consumption, and feedback on health risks (hereafter referred as “quiz”); (2) the blood alcohol content (BAC) computation module (hereafter referred as “test”); (3) the goal-setting tool (hereafter referred as “challenge”) with the possibility of obtaining a “badge” if the goal is achieved; (4) the designated driver tool (hereafter referred as “driver”); and (5) fact sheets (hereafter referred as “pedia”). The content of the app followed the existing literature [14,30-34] and previous research involving electronic interventions conducted by our group [17,18,26,27,35,36].

### Participants and Procedures

Participants were students from 4 tertiary education institutions in the French-speaking part of Switzerland (ie, higher education institutions in health: Haute Ecole de Santé Vaud [HESAV]; University of Lausanne [UNIL]; Federal Polytechnic School of Lausanne [EPFL]; and Lausanne School of Hotel Management [EHL]). To be eligible (in test 1 or 2 or both), participants had to be a student, fluent in French, own an iPhone (Apple Inc; although the final version of the app is available for both iOS and Android, the development was carried out on an iOS platform), score 4 or more (for men) or 3 or more (for women) in the AUDIT-C (Alcohol Use Disorder Identification Test – Consumption) [37,38], and provide informed consent to participate. Study promotion was conducted with the support of students’ associations or communication staff of the targeted institutions. Study presentation and contact information were displayed by flyers (displayed in the various institutions) and electronically (eg, on the students’ association page on Facebook). Interested students participated in a phone call aiming to provide information about study participation, answer all questions, and screen for eligibility. Eligible students who were willing to participate completed written informed consent and were then explained about how to download the app. As the app was in the development phase, it was not available to the public and was specifically installed on each individual smartphone. Participants then tested the app for 2-3 weeks and took part in a semistructured interview to provide feedback. During the testing period, participants received reminders to keep testing the app. Prototype 2 of the app was developed based on feedback from test 1. Issues identified by multiple participants were given priority when refining the app.

Participants included in test 1 were invited to retest the app in test 2. In addition, new participants were included to ensure a naïve feedback on the app. The inclusion criteria and procedures mirrored those used in test 1. Participants received CHF 50 (about US $50 at the time of the study) at the end of each semistructured interview. All interviews were conducted by a senior researcher (VSG), in-person, using an interview guide (see Multimedia Appendix 1). The interviews aimed at exploring the following themes: general impressions on the app, perceptions regarding the app’s usefulness, perceptions on the app functioning, perceptions on the app design, perceptions regarding the app content, perceptions regarding each module of the app, and perceptions regarding the notifications. Each interview lasted between 36 and 70 minutes. Interviews were audio-recorded and transcribed verbatim by trained research assistants. All names and identifying information were removed before analyses.

### Qualitative Data Analysis

Qualitative data were subjected to conventional content analysis. This method enables description of qualitative data through a systematic process of coding and classification [39]. Qualitative data were reviewed by VSG to identify recurring categories. Initial coding was conducted using a line-by-line technique aiming to narrate the actions occurring in the interviews [40]. After the initial coding process was completed, a codebook was created, wherein incident-by-incident codes were pooled and idiosyncratic or redundant codes were collapsed or eliminated. After the codebook was created, NB reviewed the codebook and tested it with 2 transcriptions. The codebook was then refined and finalized in consensus meetings. Finally, VSG rated all qualitative data from test 1 with the final codebook. The codebook was then adapted to rate qualitative data from test 2. Specifically, VSG reviewed a subset of interviews to identify new categories that did not appear in the original codebook. Finally, VSG rated all data from test 2. We present quotes in results from both tests 1 and 2 for illustration of categories emerging from the analysis. ATLAS.ti 7 (Scientific Software Development GmbH) was used to code qualitative data.

### Ethics Approval

All procedures were approved by the local Ethics Committee (Commission cantonale d’éthique de la recherche sur l’être humain [CER-VD]; protocol number 2018–00560).

## Results

### Test 1

Multimedia Appendix 2 shows screenshots (by module) taken at the various stages of the app development process (prototype 1, prototype 2, and final version).

#### Participants

In total, 10 students were included in test 1. Of those, despite several reminders, 1 participant did not download the app and was therefore excluded, resulting in a sample of 9 participants. The latter tested the app for at least 2 weeks and took part in a semistructured interview. The mean age of the participants was 23.11 (SD 3.76) and 44% (4/9) were female. Qualitative results identified 6 main themes as described below. See Textbox 1 for a summary of the main themes and subthemes.

**Thematic framework summarizing the major themes and subthemes.**

**Test 1**

- Theme 1: General Acceptance of the App
  General perceptions of the app
  App use in the future and recommendations to peers
- Theme 2: Importance of the Targeted and Relevant App Content
  Personalized feedback on self-reported alcohol consumption
  The blood alcohol content computation module
  The designated driver tool
  The goal-setting tool
  The fact sheets module
  Add a monitoring tool
- Theme 3: Importance of Credibility
  The cartoon character discredits the app
  The app must provide precise and valid results
- Theme 4: Importance of the App Usability
- Theme 5: Importance of a Simple and Attractive Design
- Theme 6: Importance of Notifications to Ensure App Use over Time

**Test 2**

All themes and subthemes were the same as in test 1 except for the additional following subthemes:

- Theme 2: The monitoring tool (based on feedback in test 1)
- Theme 3: The app looks more serious (based on feedback in test 1)

#### General Acceptance of the App

##### General Perceptions of the App

All participants endorsed positive perceptions of the app that was commonly described as “stimulating,” “practical,” “fun, light, and not too serious.” When describing the app, participant 2 noted: “It is not heavy. That’s what I like about it; it’s not too serious, too heavy to make you ashamed to do it.” The app was also often perceived as “interesting” and “comprehensive.” Participant 1 explained: “I found the app interesting because it brings together many things about alcohol in one single app. So there’s no need for more. I liked much the fact it was pretty comprehensive.” Furthermore, half of the participants evoked the potential impact of the app on their alcohol-related behaviors:

Every time I drank a glass of alcohol, I thought about it [the app], so I can tell it had a restrictive effect on my consumption (...). I think I did not get alcohol once or twice because I told myself “well here if you enter four times in the app it will not make it!”

Participant 3

I think it [the app] is good given that I was still able to control my consumption of alcohol. Not regarding how much I drink, but to realize a little about the level of alcohol in my blood during the period and especially if all of a sudden, I'm driving or not since I have a driving permanent license.

Participant 5

Despite a general positive perception of the app, a few participants also commented on some general aspects they did not like: 1 participant reported that he was not stimulated a lot by the app that was judged as “too discreet.” Others questioned the alignment between the app content and the targeted audience, considering that it would be better suited to individuals with alcohol problems or to younger populations:

As I felt it was more for people who thought they had an alcohol problem and wanted to evolve, rather than for people who...students for example who just need to be sensitized and see the impact that alcohol can have on them.

Participant 7

I haven't actually learned much about my consumption and the consequences, but I think for people who are younger...For example, at the age when you start drinking a little bit, you really don't realize it. I think it can be even more interesting because at my age, I'm 23 (...), I know the consequences.

Participant 8

##### App Use in the Future and App Recommendation to Peers

Most participants reported that they would still use the app after the study, most often to assess their blood alcohol level when partying. A minority of participants mentioned, however, that the app was not stimulating enough to have them using it over time. Participants 3 disclosed: “Well I think if it was really like that all the time, I would have done the process one more week and then I would have removed the app,” whereas participant 1 said: “This lack of reminders, I found it was not motivating to keep using it in the long run.”

When asking participants whether they would be willing to recommend the app to their peers, most answered that they would do so because they found the app was “fun,” “interesting,” and “easy to use.” Furthermore, participants commonly mentioned that they would recommend the app to their peers to limit the risks associated with driving while being intoxicated:

I could recommend it to them [participants’ friends], because I think it could be very useful for those who have their license, it would help them calculate their consumption (...) it's interesting to see where they stand to know if they should not drink too much...well, less drinking the time they drive and all.

Participant 4

Finally, a few participants were unsure as to whether they would recommend the app to their peers because they would not like endorsing “the moralizer role” among friends or being judged because of the use of an alcohol-related app.

#### Importance of the Targeted and Relevant App Content

##### Overview of the Content

All participants agreed on the importance to provide targeted and relevant content. Specifically, participants’ feedback on the app content consistently reflected the idea that it must be interesting, stimulating, useful, and directly beneficial to them. We describe below participants’ feedback reflecting these ideas for each module enclosed in the app.

##### The Personalized Feedback on Self-Reported Alcohol Consumption

Participants consistently mentioned appreciating the personalized feedback, commonly perceived as the most “useful,” “interesting,” “relevant,” and “impactful” part of the app. They reflected positively on the opportunity to get personalized feedback and compare one’s own drinking with their peers:

I think it's good, precisely those numbers, because that's where we really realize what we're consuming and that's when we fill out the questionnaires, well, we don't really realize if we didn't have any feedback, so I found it very interesting and that's the big positive point.

Participant 3

That's really the first incentive for me to use the app for a little...so typically with these results, I'd say that 0.5 percent of women your age drink alcohol like you do and 99.5 percent of women drink less alcohol than you do...When you get results like that, or at least when you're above average, it's a clear incentive to...well, to do something, or at least to be aware of the risks of your alcohol consumption.

Participant 1

Likewise, participants frequently found the feedback on the calorific content of the reported consumption “interesting,” “relevant,” and “impactful.” Participant 2 explained for instance:

I never think about the fact that it's very caloric and to give a number like that on a hamburger, it makes an impact because I've had several times to say to myself “well I'm not going to eat at the [name of fast food] because it's not good” and finally to realize that in one evening I may have eaten two [name of fast food], I say to myself “yeah okay.”

Similarly, most participants qualified the feedback on health risks as “interesting,” which allowed to increase awareness. Participant 5 mentioned that this feedback “allows realizing the health risks of excessive consumption.” He went on explaining that he found this particularly interesting because “you don’t really realize the problems associated with alcohol, it’s a bit of a decriminalized drug.” A minority of participants disclosed, however, that they did not read the information that was considered “too serious.”

##### The BAC Computation Module

All participants mentioned that they appreciated the BAC computation module and some of them considered this as the most useful part of the app. Most participants reported that the BAC computation module was the tool of the app they used the most frequently, generally while partying, to estimate whether or not they were able to drive home. Participant 5 reported for instance “[he] found [this part] the most useful...maybe because [he] was driving (...) and at least [he] knew that [he] could be a little safer.”

Most participants perceived the information on the risks associated with BAC as “useful” and “interesting,” whereas a minority of participants reported skimming over the reading, considering this information as “not very meaningful nor striking.”

##### The Designated Driver Tool

Most participants reported liking the designated driver tool that was frequently qualified as “funny,” “nice,” “useful,” and “practical.” Participant 2 recounted his experience while testing it: “People had a lot of fun taking pictures and then we would look at our faces in the pictures...the idea of taking pictures of everyone is fun.” The designated driver tool was also considered as potentially resolving a recurrent problem when partying. Participant 8 disclosed:

Well I think it's always the big debate when partying where nobody wants to be the driver and then even if we say “ok, ok, each one in turn”. We don’t necessarily go out with the same people every time-...if everyone get the app it's like that and not otherwise...And then at least there's no discussion (laughs).

Despite these positive perceptions, however, most participants explained that this tool did not apply to them specifically because they do not drive when partying or have already implemented alternative safe habits. For instance, participant 6 explained: “I just tried it to see what it was like, because when we go out, given that we must take a car anyway, well we decide before who will drive, well who get a car.” That said, participants commonly considered that the designated driver tool did belong to the app, considering that it might be useful in younger populations.

##### The Goal-Setting Tool

Half of the participants indicated they appreciated the goal-setting tool that was often perceived as useful to set up limits, decrease alcohol consumption, or raise awareness of one own consumption:

I think it can be pretty good because you can set a goal (...) you can even realize how much we drink because you have a little bit more perspective in this situation and say “ah, this week I actually drank more than I thought I would”, it allows you to see yourself and to realize.

Participant 5

Three participants mentioned, however, that they felt this goal-setting tool did not apply to them, perceiving it more tailored to individuals with alcohol problems. Participant 7 said for instance: “I did not really understand its usefulness (...). For me it was more for someone who think he has a problem and all of it and that he wants to achieve challenges.”

Almost half of the participants indicated they liked the badges that were perceived as “nice,” “funny,” or motivating to set up challenges. Three participants found the badges interesting without giving much importance to it, whereas another participant considered they were not useful.

##### The Fact Sheets Module

Most participants mentioned they appreciated the fact sheets module that was considered “interesting,” “useful,” and sometimes “comprehensive” or “easy to read.” Participant 4 said: “Yes, no I found it good. Additionally it is not too long, it’s good.” A few participants suggested adding topics (eg, alcohol and drugs) or making it shorter or funnier, whereas 3 participants did not like the fact sheets module, considering it as “too heavy” or not funny.

##### Add a Monitoring Module

Almost half of the participants suggested adding a module aiming at monitoring alcohol consumption over time, without the need for goal setting. According to the participants, such an addition would help increase awareness of alcohol consumption over time and could make the app more stimulating:

I would appreciate, I don’t know, at the end of the week to know how much I could have drunk or maybe where, when, maybe with whom (...). I think it might be interesting to enter this type of data. I might tend to leave it in this (current) format if I really wanted to install it. When in fact I think I would be quite assiduous... it would already be interesting to know the results over a month, I think.

Participant 3

And I would have liked to have, in relation to the evolution over time, but over a longer period of time, a kind of graph that would follow us over time as well. I would also have found it interesting to see more like that because well, the app is good... Well, yeah, I couldn't figure out what I was consuming more than that either.

Participant 7

#### Importance of Credibility

##### The Cartoon Character Discredits the App

**Figure 2:**
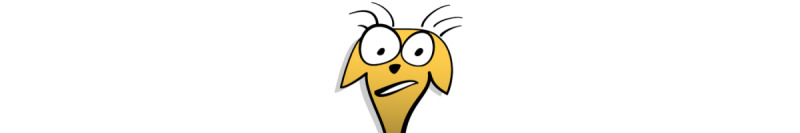
The abandoned character included in prototype 1.

All participants made comments highlighting the importance to make the app look serious and credible. In prototype 1, a cartoon character was included to guide participants through the app (Figure 2), a feature that has been used successfully in other electronic interventions [41,42].

The most consistent comment was related to this character that was commonly perceived negatively. Only a minority of participants reported they liked the character that was described as “funny,” “nice,” and “lightening the app.” Most participants explained they did not approve of the character that was considered “childish,” not tailored to the content and population targeted by the app, and ultimately at risk to discredit the app:

Now, I'm not a big fan of the drawings themselves (...) I find it a bit childish, too childish. I find the idea of using character makes it lighter, but it makes it too light and I feel like it's a child's application when it's not at all and it's almost out of context (...) it looks like they're taken from a child's comic book and I don't find it logical. It makes the application a bit less credible.

Participant 2

After that, it's... a small criticism, it's also the logo. I don't know if it's tailored to a consumption... it's a little bit very childish. (...) and isn't there a refusal to use an app where the logo is too childish because young people are not taken seriously. Maybe in their minds it's a bit of a refusal.

Participant 5

Relatedly, participants commonly described the icon of the app (ie, displaying the face of the character) as “childish” or even “ridiculous.” Consequently, most participants recommended removing this character from the app and the icon.

##### The App Must Provide Precise and Valid Results

Almost half of the participants questioned the normative feedback results’ validity, assuming that they were computed with data gathered by the app over time. Using data from serious research was perceived as necessary to consider and value the normative feedback results. When providing feedback on the normative feedback results, participant 2 explained: “I thought it wasn’t worth much because it came from....in comparison with other people using the app (...), whereas if I know that it comes from serious statistics, it will worth more to me.” After receiving an explanation related to the data used to compute the normative feedback, participants consistently recommended making this more visible in the app to increase the perceived normative feedback results’ value among future users (eg, adding a pop-up after data completion or highlighting the research reference used to compute the normative feedback).

Furthermore, participants commonly questioned data taken into account to compute the BAC, reflecting a common expectation for accuracy and precision. Most participants mentioned that the BAC computation was approximate considering other influencing factors (eg, precise number of consumption hours, having eaten or not) were not accounted for.

Relatedly, entering alcohol use data with the use of standard drinks was perceived as “too vague.” Participant 7 explained, for instance, that he found the questionnaire complex to fill in correctly the because of the alcohol concentration differences across beverages:

Filling in 100% right was complicated. (...) I like Belgian beer, which is often a bit strong (...) it's a beer with more than eight percent, it's not the same thing (as a regular beer). If I fill beer I'm not in the right category. So... and it's the same at that point it's also a bit complicated because what do you do? I still have my blood alcohol level, which is still double that of a standard beer.

#### Importance of the App Usability

All participants agreed on the fact that the app was globally easy to use. They commonly qualified the app as “clear” and “intuitive,” which was outlined as an essential ingredient to be further used. The fact that the app did not require time nor reflection to use it was also commonly highlighted as positive and important. Participant 1 said: “It is simple and straightforward, no questions, and this is really good. In that, well, the easier, the more effective, the better.” Relatedly, participant 6 appreciated its interface because it was easy to use:

You can see very well what it is used for. As you open it, you can see that you have the different options between the Quiz, to choose your driver, whether it's to get information. So that's good, I mean it's visible and it's clear what we can do.

In parallel, participants demonstrated very little perseverance when facing use challenges. Most difficulties involved entering alcohol consumption data in standard drinks. Participants commonly mentioned that they were unsure when filling in the alcohol questionnaire, most often because they hesitated about which category fits their drinks best. To address this difficulty, participants suggested adding information describing the categories and making it obvious to find. Participant 2 suggested: “Putting the ‘i’ [relating to information] near the alcohol categories to really explain what these categories correspond to, well to put the ‘i’ there because that’s where I would have looked for it.” Finally, a few participants reported difficulties to understand their normative feedback and suggested to make the feedback more visual and to simplify the sentences.

#### Importance of a Simple and Attractive Design

Only a minority of participants mentioned they liked the design, evoking the fact that it was not too serious or that the colors fit well. By contrast, participants commonly disliked the design, commenting it as “obsolete” and “not visual enough.” Similarly, the icon was frequently perceived as “nonvisual,” “not nice,” or “nonfinished.” Participant 1 said, for instance, that he “had the feeling to see a 2 or 3-year old app regarding the interface,” whereas participant 9 mentioned that “he found the lay-out not friendly” and not achieved, assuming it was related to the fact that the app was not over yet.

Consequently, participants recommended improving the design to make it more attractive while making it simple and sober. Participant 8 suggested for instance: “Well it could be a little more visual. I don't know with different colors (...) or maybe a gauge with...if you’re in a non-risk situation in green and a risk situation in red,” whereas participant 5 disclosed: “It can be just something pretty elegant, simple, like a university campus app.”

Finally, similar comments applied to the badges included in the goal-setting module. Participants commonly disliked their design, considering they were “childish,” “too complicated,” and “not clear.” Participants explained they did not understand the meaning of the badges while looking at them, nor the association between the drawing and the meaning of the badge. Therefore, they recommended using more simple drawings and displaying them in a logical order to ease the understanding and make this part funnier and more stimulating.

#### Importance of Notifications to Ensure App Use Over Time

Participants generally mentioned that they found the notification frequency acceptable, although a minority suggested increasing its number. Participant 3 expressed being surprised to receive few notifications, which could have decreased the app use over time: “I was expecting to be more stimulated by the app (...) so I could have forgotten about it (...) without being solicited so I could have put it aside.”

Half of the participants considered that the notifications they received while testing the app were useful to remind them using the app and decrease the risk to forget the app:

So I thought it was nice because sometimes it's true that with the days that go by and everything is forgotten and sometimes I had the little notification and I thought “ah, I have to go and take a look at the app.”

Participant 4

Participants expected receiving notification to remind them to fill in their ongoing goal-setting challenge. Participant 1 explained he “did a 7-day goal-setting challenge and found surprising not receiving a reminder.” Hence, they recommended adding notification with specific content, most often to remind them fill in the ongoing goal-setting challenge. Corroborating previous comments, adding notification was perceived as essential to fill in data over time:

It’s true that these (goal-setting) challenges I forgot to fill them and then I gave up two or three of them because I forgot to fill them. Now it might be nice to get a notification that...so “today, don’t forget to fill in your consumption in the challenge.”

Participant 6

### Modifications Based on Test 1 Qualitative Findings

The major modifications concerned the design (change of name, icon/logo, home menu, general presentation, clarification of the alcohol use questionnaire with more specific options for standard drinks, presentation of feedback parts [normative feedback and BAC]) and the development of a new module allowing day-to-day monitoring. References for the normative feedback were added or placed in more prominent display to support the app’s legitimacy. A more readily available and more detailed description of standard drinks was included in the app with an option to access more detailed information on standard drinks. Different drinks choices were added. For example, an option for stronger beers/large beers (equivalent to 2 standard drinks) was added. The cartoon character was removed from the app’s icon. Similarly, the cartoon character that was created to help users navigate the app was removed. Within-app notifications and connecting messages (via within app pop-up messages or messages at the end of the feedback) between modules were included (ie, prompt to set drinking goals after an assessment of one’s alcohol use, indications on the evolution [increase or decrease] of one’s alcohol use since last use). How to obtain the different badges in the goal-setting module was also added (available when users click on any given badge). Table 1 presents how these badges can be earned. The full series of potentially collectible badges was displayed (with to-be-obtained badges grayed). Notifications and visual prompts were modified (notably, a red dot announcing that one should report something).

**Table 1:** List of the 15 badges and how they are earned^a^.

Badge type	Challenge
Badge 1	For the first completed challenge
Badge 2	For drinking exactly the amount of the goal (set by the user)
Badge 3	For drinking less than the goal on any challenge
Badge 4	For drinking half the amount (or less) of the goal
Badge 5	For drinking no alcohol during a 1- or 2-day challenge
Badge 6	For drinking no alcohol for 1 week
Badge 7	For completing 2 challenges in a row and drinking less than half of the goal
Badge 8	For completing 2 challenges in a row with no drinking
Badge 9	For completing 3 challenges (equal consumption)
Badge 10	For completing 3 challenges (lower consumption)
Badge 11	For completing four 7-day challenges without any alcohol
Badge 12	For completing 5 challenges
Badge 13	For completing a challenge during Saint Patrick’s Day
Badge 14	For completing a challenge during Christmas
Badge 15	For completing a challenge during New Year’s Eve

^a^For each challenge, the goal (ie, drinking limits) and duration of the challenge are decided by the user. The goal can be over the recommended drinking limits.

### Test 2

#### Participants

The 9 participants who took part in test 1 were invited to test prototype 2 of the app. Of those, 6 accepted, whereas 3 declined because they were not available at the moment of the test (ie, all 3 were temporarily outside of the country). In addition, 5 new students were included in test 2, resulting in a sample of 11 participants. A comparison of the qualitative inquiry at test 1 between the 6 participants who accepted to take part in test 2 and the 3 who declined revealed no distinct trends differentiating their perceptions of the app. Semistructured interviews were conducted in-person with interview guides (see Multimedia Appendices 3 and 4). The mean age was 32.1 (SD 4.38) and 55% (6/11) were female. Qualitative results mirrored the main themes that emerged in test 1. Each theme is described below.

#### General Acceptance of the App

##### General Perceptions of the App

All participants—from test 1 and the new ones—spontaneously evoked at least once liking the app that was commonly described as “fun,” “interesting,” “not too heavy nor complicated,” “not moralizing,” “welcoming,” and “not boring.” Similarly, most participants from test 1 spontaneously mentioned they found the app improved, most often with regard to its design. Participant 2 said for instance:

I’m glad to see that the comments were taken into account and that it was...On the one hand so much better, but I noticed it especially on two points when I opened on the design level, it was especially on this point that I had insisted a lot. And it's really changed a lot and I think it’s good.

As many as 4 out of the 5 new participants evoked the potential impact of the app on their alcohol-related behaviors. Participant 10 (new) disclosed for instance:

I was at a restaurant and I hesitated to have a beer and I thought “Ah I’ll have to put it in the app, that's silly!” And so I didn't take it.

##### App Use in the Future and App Recommendation to Peers

In line with findings from test 1, most new participants (3 out of 5) and those from test 1 reported they were willing to continue using the app after the study most often to help maintain awareness on their drinking. Participant 7 explained, for instance, that he “would keep using the monitoring to see how he drinks over a longer period of time.” In the same line, when asking new participants whether they would be willing to recommend the app to their peers, all answered they would do so generally because it could enable them being aware of their drinking or decrease alcohol-related risks when partying.

#### Importance of the Targeted and Relevant App Content

##### Content Overview

In line with results from test 1, all participants highlighted the importance of providing interesting, stimulating, and useful content.

##### The Personalized Feedback on Self-Reported Alcohol Consumption

Consistent with this idea, 4 out of the 5 new participants mentioned liking the personalized feedback that was perceived as “interesting,” useful to compare one’s alcohol use with peers, and even the “added value of the app” for some of them:

It’s the first time I’ve seen it [the personalized feedback] in an application like this (...). I think it’s a very good idea. Because it gives a first feedback to say...with numbers, (...) it can either give a slap to say “Ah well look how you’re consuming”, or on the contrary, it can be “Ah ok, that’s fine.”

Participant 12, new

Likewise, new participants commonly perceived the feedback on calorific content of the reported consumption as “interesting,” “fun,” and “punchy.”

##### The BAC Consumption Module and the Designated Driver Tool

In the same vein, 4 out of the 5 new participants mentioned that they appreciated these modules and the information on the risks associated with BAC that was considered “useful,” “interesting,” and “informative.” Participant 11 reported, for instance, that he found the BAC computation tool was useful to set up drinking limits when one drives.

##### The Goal-Setting Tool

As many as 3 of the 5 new participants reported they liked the goal-setting tool, most often because it was considered “useful.” Participant 13 disclosed for instance:

I like it a lot and I thought it was great because it makes you realize how much you're drinking and at the same time, you can say: “Yeah next week I’m trying to have one less drink, see how I feel, see if it's going to change something at my party, change something in my body.”

On an interesting note, 2 participants (1 from test 1 and the other new) suggested to add a 1-month goal-setting challenge in the tool. In line with findings from test 1, however, 1 participant felt that this module was more tailored to individuals with alcohol problems instead of students and half of the new participants mentioned they did not use the goal-setting tool because they found it was not useful to them.

All new participants showed interest in the badges, perceived as “fun,” “motivating,” and “rewarding.” However, 2 of them questioned the choice of badges’ names, perceived as “meaningful” or not directly related to the badge content. Relatedly, using English words and referring to foreign holidays (eg, Saint Patrick’s Day) were questioned, highlighting the importance of relevant content tailored to the target population.

##### The Monitoring Tool

Most participants from test 1 and 4 out of the 5 new ones showed interest in the monitoring tool. Almost half of the participants mentioned that this tool was the best part of the app. The monitoring was perceived as “relevant,” “impactful,” “interesting,” and “useful.” Participants commonly evoked that they appreciated following their alcohol use over time. Likewise, the related statistics were perceived as “interesting,” “meaningful,” and “impactful”:

Finally the section I preferred is the monitoring. To indicate your consumption without necessarily giving a constraint it just allows you to see it written and to see “Ah, today I drank so much, today I drank so much. I say to myself,” “Ah, maybe I drank a little too much.”

Participant 12

And what I liked is that we could have the statistics. So that’s really good because you can see between weekends or between certain times when if all of a sudden we increase our consumption or if we decrease it or if we are a little stable, I thought that was pretty good.

Participant 4

A few participants mentioned, however, that recording drinking is “constraining” and requires assiduity and that only motivated users might keep monitoring their alcohol use over time. To help address this issue, participants consistently recommended adding notifications.

##### The Fact Sheets Module

In line with results from test 1, new participants consistently mentioned they appreciated the fact sheets module perceived as “comprehensive,” “interesting,” and “easy to read.”

#### Importance of Credibility

##### The App Looks More Serious

As many as 4 of the 6 participants from test 1 spontaneously reported that they found the design improved because it was “less childish” and “more tailored to the target public.” The 2 other participants from test 1 described the design as “more serious” and “less fun”; they noted that they liked the light design from the previous version while finding the new one acceptable. Participant 3 disclosed for instance:

I though it [the design] was already good because it wasn’t too serious. Now it’s a little more serious but it doesn’t bother me at all because you still need to be credible for the application. So yeah I think it was a good idea to change the name and logo.

##### The App Must Provide Precise and Valid Results

Similar to test 1, participants commonly highlighted the importance of precision and validity in the app content. Participant 3 explained, for instance, that “when using this kind of app he wants to make sure to get convincing and reliable findings.” The most common comment in this regard was related to the BAC computation module. One of the new participants commented that he used another calculator to verify the results, leading to slight differences, which was perceived as potentially “discrediting the app.” Relatedly, other participants (new and from test 1) suggested ways to improve precision of the results, such as “providing the possibility to indicate the precise time of consumption” (ie, times of the first and last drinks), or adding an option to account for food consumption.

Other comments questioning the precision of the findings were related to the questionnaire measuring alcohol consumption. Some participants suggested adding the possibility to indicate alcohol percentage included in the drinks instead of indicating the beverage itself to improve measurement precision. Furthermore, the item measuring typical alcohol use per day (ie, “on a typical day, how many drinks do you drink?”) resulted in understanding issues in half of the participants from tests 1 and 2. Participants commonly reported being unsure about how to answer this question correctly, potentially leading to unprecise answers. Participant 2 suggested improving the item to make it clearer:

Does that mean that if I’m used to drinking (...) we’ll say 7 beers but only once a week, should I put that I drink 1 beer a day every day or should I put that on a drinking day I drink 7 beers. It’s really not clear to me. For me it's the “usual” that’s wrong. (...) in my opinion it would make more sense to write down on a drinking day, how much alcohol do you drink? In any case, to make that more explicit.

Finally, unlike test 1, participants did not question the validity of the personalized feedback results. Some participants from test 1 noticed some improvements in this area in the new version:

I was happy to see that you took the comment I made into account, that we didn’t know where those numbers came from. Because the first time I didn’t know whether it was in relation to other users of the application or in relation to statistics that came from somewhere. So now it’s clear.

Participant 2

#### Importance of the App Usability

Echoing results from test 1, all participants perceived the app as globally easy to use, commonly described as “clear” or “intuitive,” which was perceived as important. For instance, referring to the BAC computation tool, participant 13 said: “It is something very quick to do. Hop, hop and tac and it is done, which I found good.” In the same vein, the monitoring tool was commonly perceived as “easy to use.” Three participants suggested linking the different modules enclosed in the app to avoid users filling in their alcohol consumptions several times. Participant 1 explained for instance: “if I take the time to fill up my blood alcohol level for example, or a challenge, it can be interesting that it is directly found in monitoring.”

In parallel and consistent with findings from test 1, participants demonstrated little perseverance when facing use difficulties and recommended to make things obvious and clear wherever possible. The goal-setting tool caused the most difficulties in use. Participants were commonly unsure about how to use it and confused regarding the meaning and use of badges:

You answer but you don’t know which one...well, if you answer a challenge, if you validate a challenge, well, I don’t know which badge I get. Maybe it’s written underneath the badges, but I admit I didn’t make the effort.

Participant 3

In response, participants recommended to make this module clearer by adding information to guide users more efficiently.

#### Importance of a Simple and Attractive Design

The perceptions of the design were globally more positive in test 2 than in test 1. As many as 4 of the 6 participants from test 1 mentioned that they found the design was much improved, qualified as “more mature” or “professional.” Similarly, perceptions of the design were positive among 4 of the 5 new participants who described it as “simple,” “clear,” or “efficient” (the fifth new participant perceived the design as “too basic”). Unlike in test 1, the modified icon of the app was endorsed by most participants who described it as “much better than in the previous version,” “clear,” or “fitting the app content.” Similarly, most participants appreciated the new version of the personalized feedback, which was commonly considered “easy to understand,” “visual,” and “clear.”

Although perceptions of the design were globally more positive than in test 1, participants made several recommendations to improve it. Participants (from test 1 and new ones) recommended, for instance, adding more colors in general and using pictures illustrating beverages in the alcohol questionnaire to make the design more attractive:

In the questionnaires and in the choices of the types of alcohol we drink (...) what could perhaps be better in my opinion and a little more fun would be to use pictures instead. Maybe a little more illustrations again. You see it’s really kind of something that people like, to have a visual aspect in this app, a little bit colorful and all that. (...) That it’s more eye catching.

Participant 14

Similarly, almost half of the participants recommended making changes in the app home screen that was qualified as “unattractive” and “too basic.” Improvement suggestions included rounding the corners of rectangles, using icons instead of white bars, and again, using more colors and pictures.

#### Importance of Notifications to Ensure App Use Over Time

Consistent with results from test 1, participants expected receiving text notifications to remind them to fill in their ongoing goal-setting challenges. Similarly, participants commonly recommended adding text notifications to remind users to fill in the monitoring:

It was after I registered a challenge, I didn’t get a reminder. I had done a kind of diet, I had downloaded an app that asks for everything you eat (...) it would write to me every time after a meal “Don’t forget to tell us about the meal” And finally it’s true that I would say to myself “Ooh I forgot to fill in” and then I would go. well I like to be reminded because I tend to forget.

Participant 13

I realized that for the monitoring, it is noted in red and I did not receive any notification (...). I almost wish I had a notification that said during the day “record your consumption” so that I would remember to do it. (...) Because it’s actually within the app and if I don’t go there every day, I don’t see it. Except that I don’t think about going there every day. It’s true that if you want to monitor, it would be nice to have a notification per day, since it’s a daily analysis.

Participant 7

### Modifications Based on Pretest 2 Qualitative Findings

Further modifications were made on the design with modifications to the home page (icons added), ordering of modules, graphical presentation of the normative feedback results, and presentation of the monitoring data. Data entered in the monitoring module were transferred into the goal-setting module (if activated) and vice versa. The structure of the fact sheets module was modified. A 1-month challenge option was added. Presentation of the BAC calculator results was updated with presentation of possible symptoms at the BAC reached for the reported consumption. Because of financial constraints and availability of numerous calculators, we did not include the possibility to report food intake as part of the BAC computation. Screenshots of the final app are presented in Multimedia Appendix 2.

## Discussion

### Principal Findings

We developed a smartphone app targeting unhealthy alcohol use for students using an iterative development process. This study indicates that a smartphone app is an acceptable way to deliver unhealthy alcohol use interventions to students, a population with notable technology skills. Although the app was generally well accepted and appeared to be a suitable mean to deliver a brief intervention for unhealthy alcohol use, qualitative interviews allowed us to identify important aspects for the target audience: the app has to have a high level of usability, its design must be simple and attractive, users must consider the app content targeted and relevant to their needs, the app should have a high level of credibility, and finally, notifications and prompts are crucial to keep users interested and engaged. Through the iterative process we were able to develop an app that incorporated evidence-based elements from other electronic interventions and that corresponds to the needs and perceptions of the targeted audience. Our results inform on app development and the need to focus on elements of relevance and scientific credibility, as well as clear and effective presentation of scientific data. In addition, independent of its content, an app has to present a high degree of usability (ie, any task required by the app that is not immediately understandable will be abandoned) and an up-to-date and targeted design. Any feedback must be easily and immediately interpretable (hence the necessity to present scientific data on readily understandable graphic format). The app should also be adapted to the local and cultural context and some features, such as a cartoon character, may be well received in some but not in other populations groups. Similarly, especially among students, some of whom may be studying computer programming and app development, there is a high sensitivity for an up-to-date design, which may limit the half-life of apps.

Currently, the evidence for efficacy of a smartphone app for unhealthy alcohol use is scarce and thus there is an urgent need for efficacy data. A key feature in being able to assess an intervention’s efficacy is giving the studied intervention the best chance to be used by its target population [43]. One of the challenges of electronic interventions is to have users involved with the intervention content, notably with repeated use of the app. This study adds that by involving members of the target population in the development of the intervention, substantial modifications can be made to the design and presentation of its components. These changes should lead to a more targeted app design. As an example, comments were made on the prototype 1 app icon which, for some users, would have led to a lack of interest in the app before even assessing its content. As such, design plays a crucial role in electronic interventions’ abilities to reach their target audience. While this qualitative study did not lead to changes in terms of the evidence-based components of the intervention, major changes were made on how the feedback was delivered, how results were presented, and how people would perceive the app, notably in terms of scientific credibility, noting that, in the studied population, the app developed by a university hospital was not sufficient and that data sources (eg, for the normative feedback) had to be prominently displayed to increase credibility and relatedness to the feedback results.

### Limitations

This study presents limitations. A notable limitation is linked to its own justification: while we targeted a population to develop a specifically suited intervention (ie, students from tertiary education institutions in Switzerland), the results are only relevant to this specific population and generalizability is limited. Nonetheless, broad categories are identified that are likely relevant to other populations as well. Other studies have shown the importance of credibility, ease of use, and tailored content [30,44-46] and while achieving this may differ according to different context and populations, we expect it to be relevant for other populations than just for the sample studied. As participants were specifically recruited to test the app, our study sample comprises people who were likely more motivated than the target population intended to use the smartphone app. Thus, results from this study will have to be compared with usage data that will be obtained in the second phase of the study (randomized trial).

Although our results are informative on the development process of the app, we currently do not have data on the app’s effectiveness in reducing unhealthy use of alcohol nor on how and when the app is used. This will be the subject of the ongoing randomized trial (started in May 2021).

### Comparison With Prior Work

Our results bear similarities with other studies on electronic interventions. Notably, Baumel and Kane [30] have shown the importance of design efficiency in real-world usage of eHealth interventions. In a study on a smartphone intervention targeting drinking conducted among members of the British Armed Forces, Puddephat and colleagues [45] showed the importance of credibility of information, targeted and personalized content, ease of use, and simplicity [45]. Garnett and colleagues [31] also showed the importance of the credibility of scientific sources. In a usability testing study among young adults who participated in focus groups after using a smartphone app targeting harmful drinking, Milward and colleagues [44] showed the importance of design, ease of use, and tailored content. Outside of a research setting, available evidence indicates that users will choose an app based on its look and credibility and tailored content [47].

### Conclusions

This qualitative study conducted among students shows that smartphone apps targeting unhealthy alcohol use need to have a simple and attractive design, tailored features, scientific credibility, be easy to use, and that the app should regularly send notifications.

## Appendix

Multimedia Appendix 1 Interview grid, test 1.

Multimedia Appendix 2 Screenshots, by module, of the app at the various stages of the development process.

Multimedia Appendix 3 Interview grid, pretest interview 2, group 1 (pretest participants 1).

Multimedia Appendix 4 Interview grid, pretest interview 2, group 2 (new participants).

## Abbreviations

AUDIT-C: Alcohol Use Disorder Identification Test – Consumption
BAC: blood alcohol content
EHL: Lausanne School of Hotel Management
EPFL: Federal Polytechnic School of Lausanne
HESAV: Haute Ecole de Santé Vaud
UNIL: University of Lausanne
