# Supplementary material for: Development of a Secondary Prevention Smartphone App for Students With Unhealthy Alcohol Use: Results From a Qualitative Assessment
Source: JMIR Hum Factors. 2023 Mar 7;10:e41088. doi: 10.2196/41088 (PMC10031438; doi:10.2196/41088)
Supplement: Multimedia Appendix 1 [file humanfactors_v10i1e41088_app1.docx]

| **Appendix 1 : INTERVIEW GRID**  Test 1 |
| --- |

| **THEMES** | **QUESTIONS** |
| --- | --- |
| **General impressions**  **Usefulness** | What do you think of the application?   - What did you like most about this application? - What did you not like about this application?   What marked you? Why?  To what extent would you recommend the application to your friends, why?  What did you find useful in this application?  What didn't you find useful in this application?  How much do you expect to continue using the application? |
| **Ergonomics in general**  **Operation**  **App functioning**  **Application design**  **Content of the application**  **Module not used**  **Module drop out** | How was your experience with the application?  What do you think of the way the application works?  What difficulties have you encountered?   - Which module(s)? - Bugs ?   How easy was it to find what you were looking for (not the content but the location within the application)  What do you think we could do to make the application easier to use?  What do you think of the design of the application in general?  What do you think of the character?  What do you think of the content of the application?   - Modules used/preferred, why?   *If one of the modules has not been used, bring print screens and evaluate with the person during the interview:*   - Why not use it? - What could be improved to make it more attractive to use?   *If there are dropouts (significant number) in any of the modules, bring print screens and evaluate with the person during the interview:*   - Why abandoned? - What could be improved to make it more appealing? |

| **Evaluation of each module of the application** | |
| --- | --- |
| **Quiz**  **Challenge**  **Test**  **Driver**  **Pedia** | What do you think of the Quizz?  What do you think of the feedback?  What was your reaction when you first read the feedback?  What do you think of the recommendations made at the bottom of the feedback?  According to the indicated consumption, Meerly proposes to make a challenge  What do you think?  What do you think of the badges (*show page if necessary*)?  What do you think of the results (*show page if necessary)*?  What do you think of the statistics (related to your challenges)  What do you think of the Test?  What do you think of the results?  Have you read the health risks posted below the BAC level?  What do you think?  What do you think of this module?  What do you think of this module?  Which pages did you find interesting and why?  What part(s) did you not like about this module and why?  What information do you think we should add/delete? |
| **Various** | |
| **Notifications** | What do you think about notifications?  Which notifications have you noticed? Why did it happen?  How useful do you think notifications are?  What notifications could we add? |
| **End of the interview** | |
| **Any additional information** | Is there anything else you would like to share before we end this interview? |
