# Supplementary material for: Development of a Secondary Prevention Smartphone App for Students With Unhealthy Alcohol Use: Results From a Qualitative Assessment
Source: JMIR Hum Factors. 2023 Mar 7;10:e41088. doi: 10.2196/41088 (PMC10031438; doi:10.2196/41088)
Supplement: Multimedia Appendix 2 [file humanfactors_v10i1e41088_app2.docx]

Screenshots from the development phase (prototype 1 and 2) are in French as the app was developed in French before being translated in English for the final version (final version available in French and English). When no changes were made between the prototype 2 and the final version, screenshots of the final version are shown.

| **Home page** | | |
| --- | --- | --- |
| **Pre-test 1 (prototype 1)** | **Pre-test 2 (prototype 2)** | **Final version** |
| 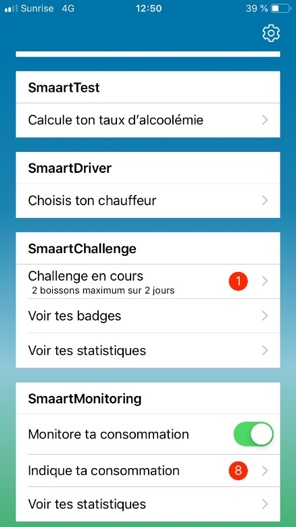 | 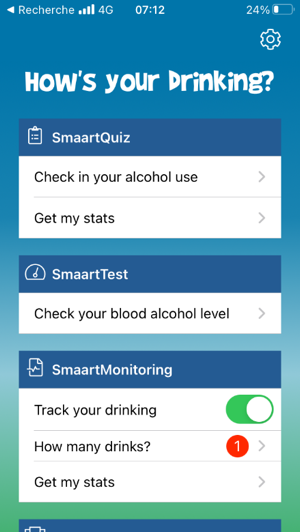 | |

Figure 2 continued

| **Personalized feedback (Quiz)** | | |
| --- | --- | --- |
| **Pre-test 1 (prototype 1)** | **Pre-test 2 (prototype 2)** | **Final version** |
| 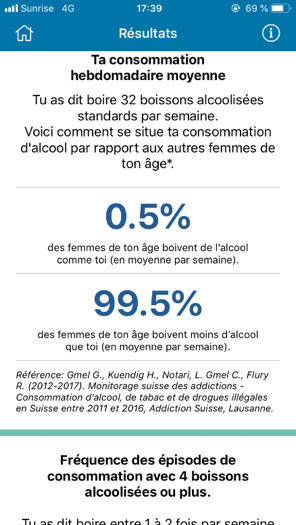 | 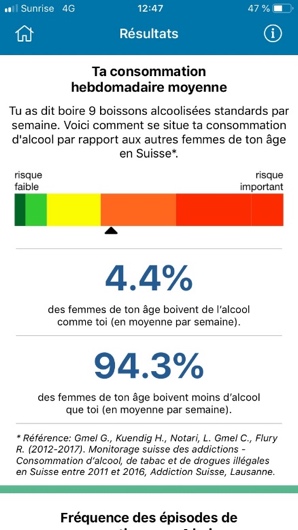 | 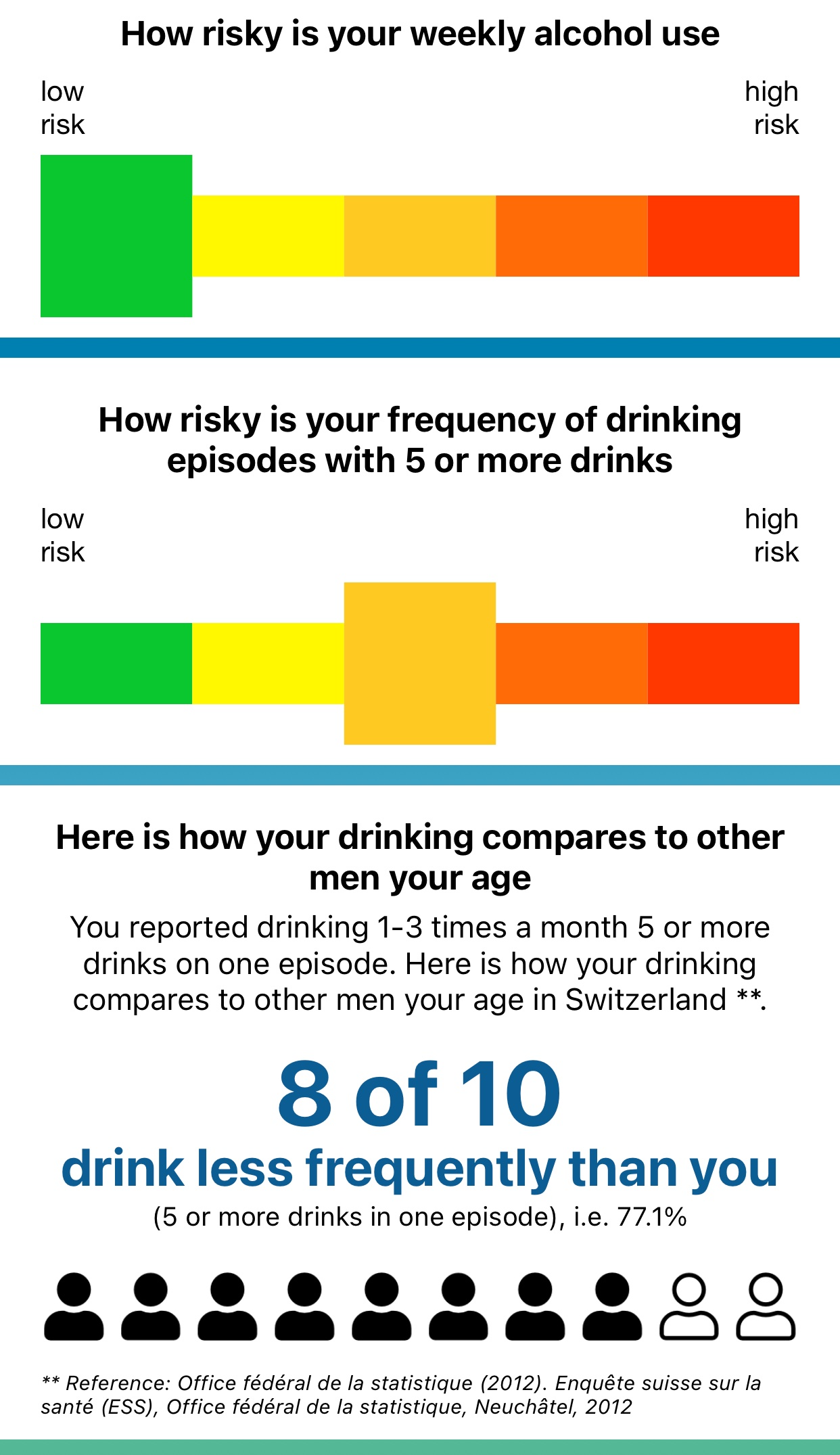 |
| 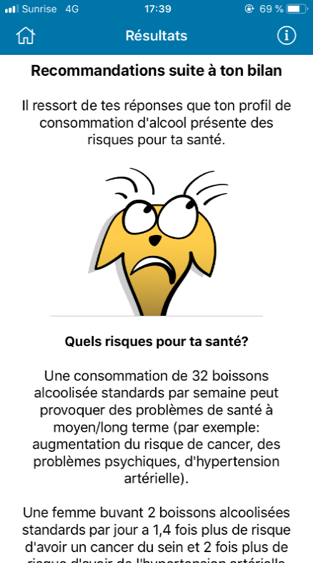 | 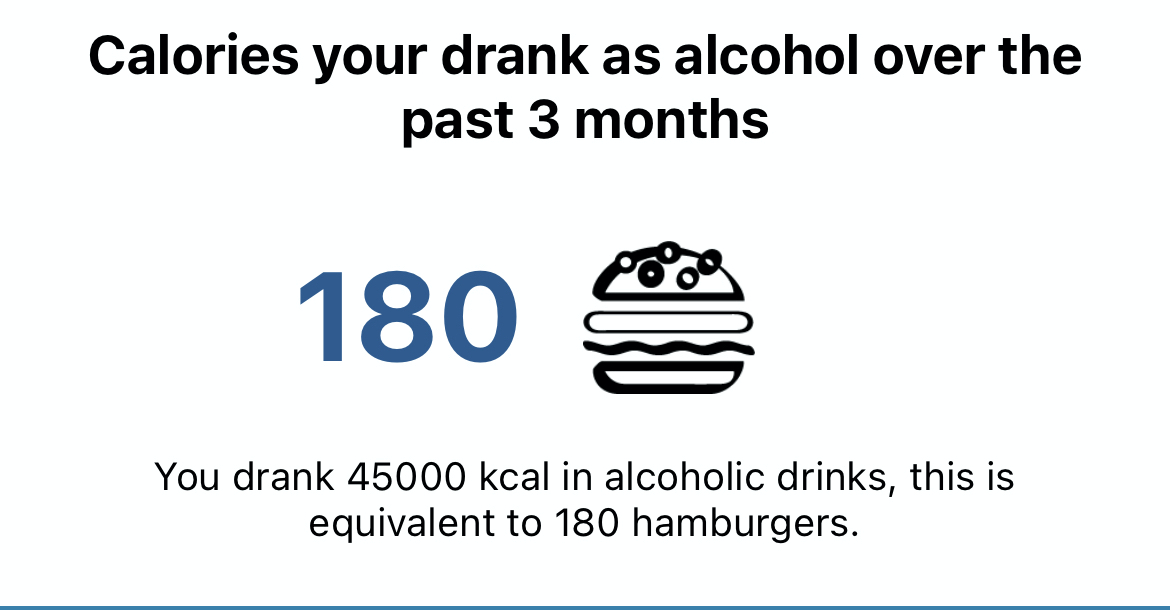 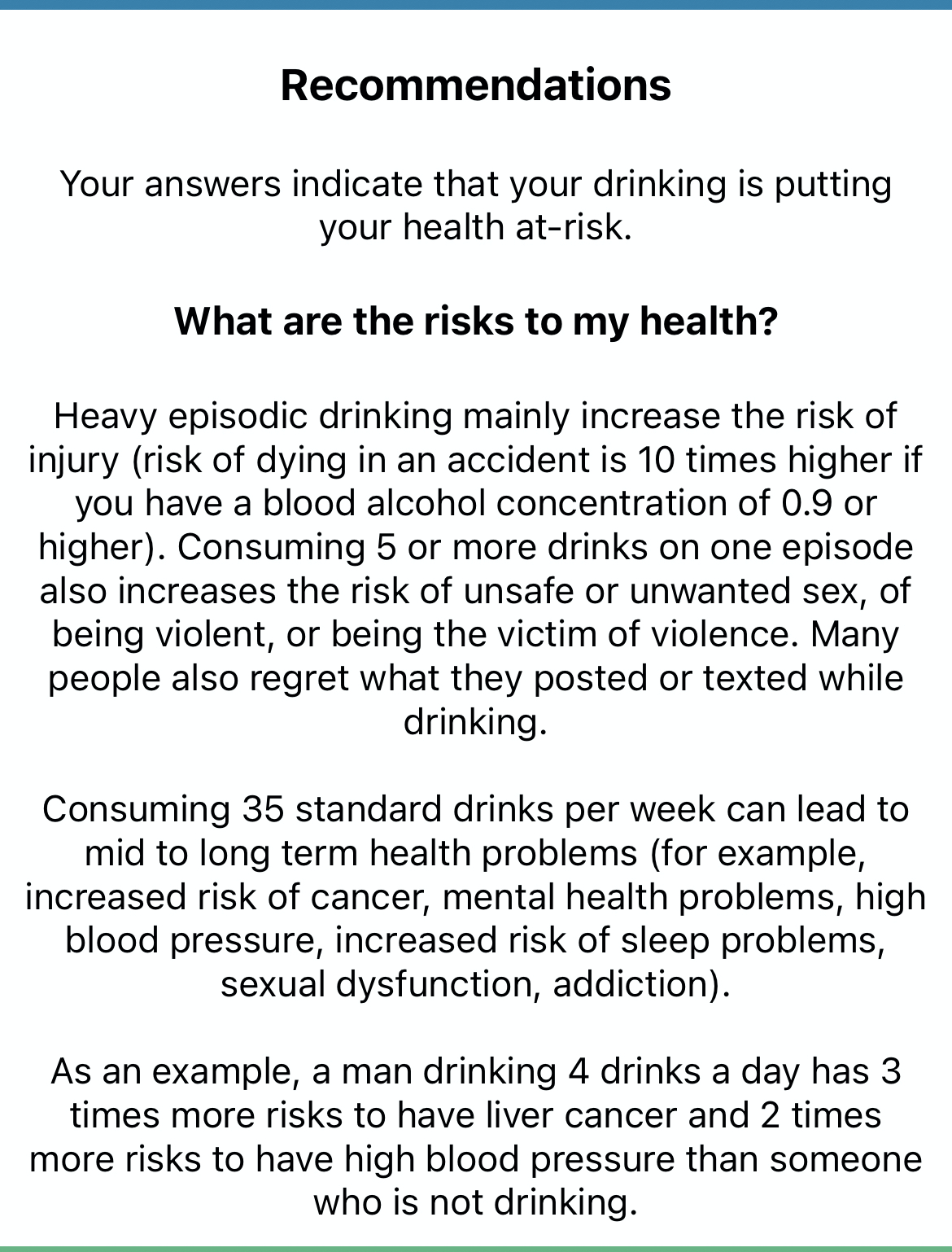 | |

Figure 2 continued

| **Blood alcohol content computation (Test)** | | |
| --- | --- | --- |
| **Pre-test 1 (prototype 1)** | **Pre-test 2 (prototype 2)** | **Final version** |
| 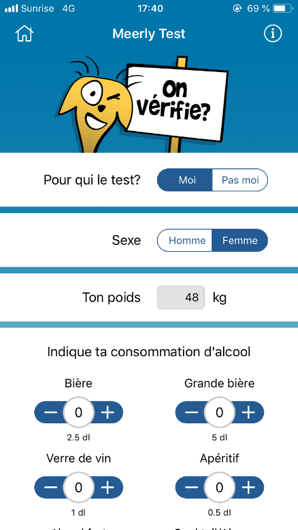 | **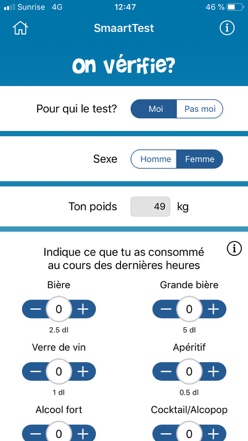**  When reporting their alcohol use, users can access the definition of a standard drink by clicking on the “i” | 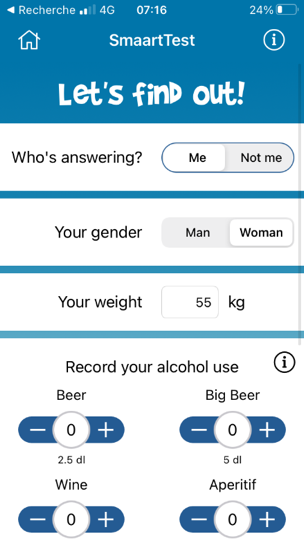 |
| 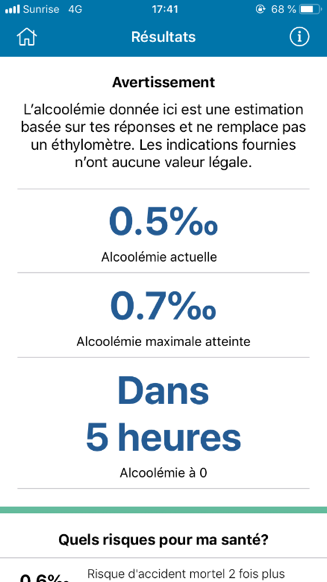 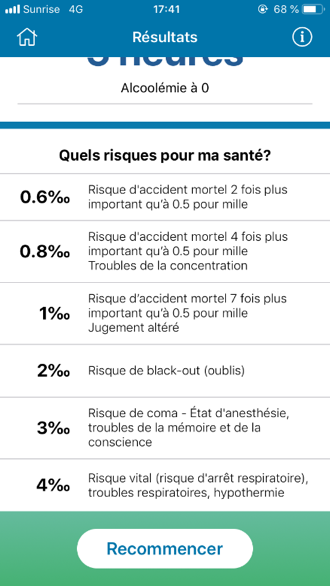 | 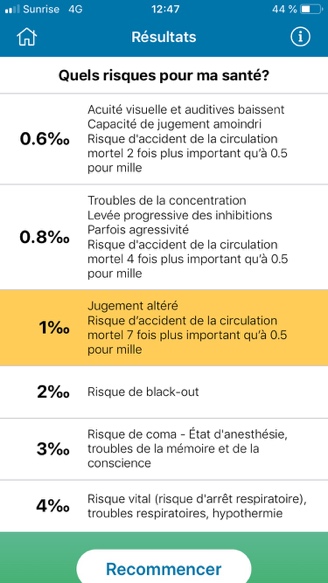 | 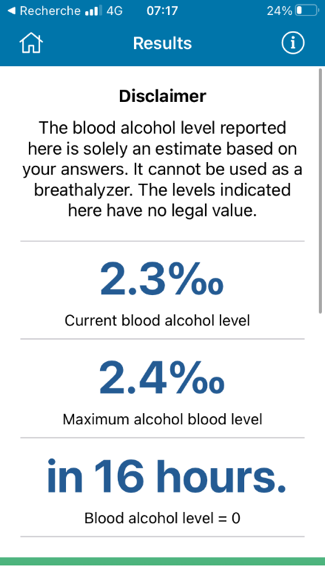 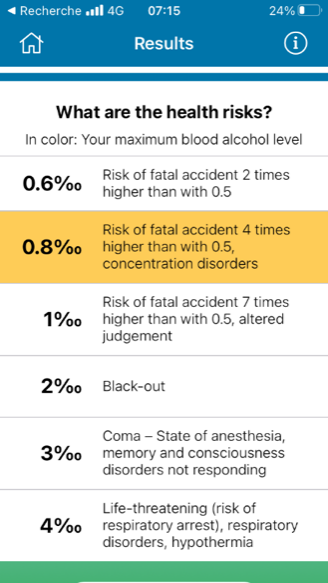 |

| **Monitoring** | | |
| --- | --- | --- |
| **Pre-test 1 (prototype 1)** | **Pre-test 2 (prototype 2)** | **Final version** |
| **Not developed** | 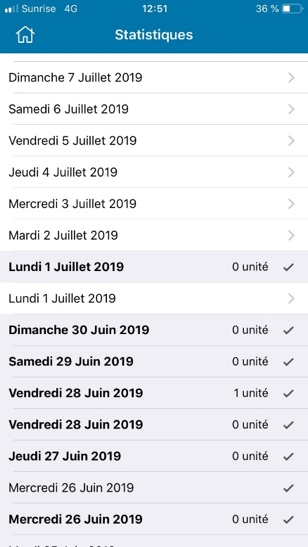 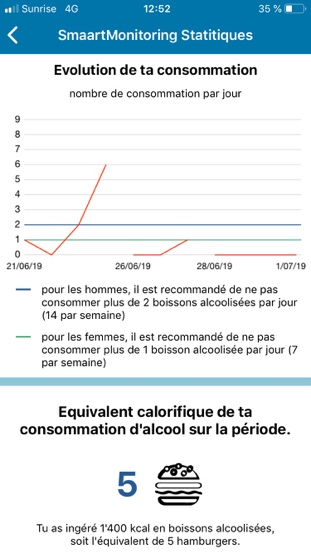 | **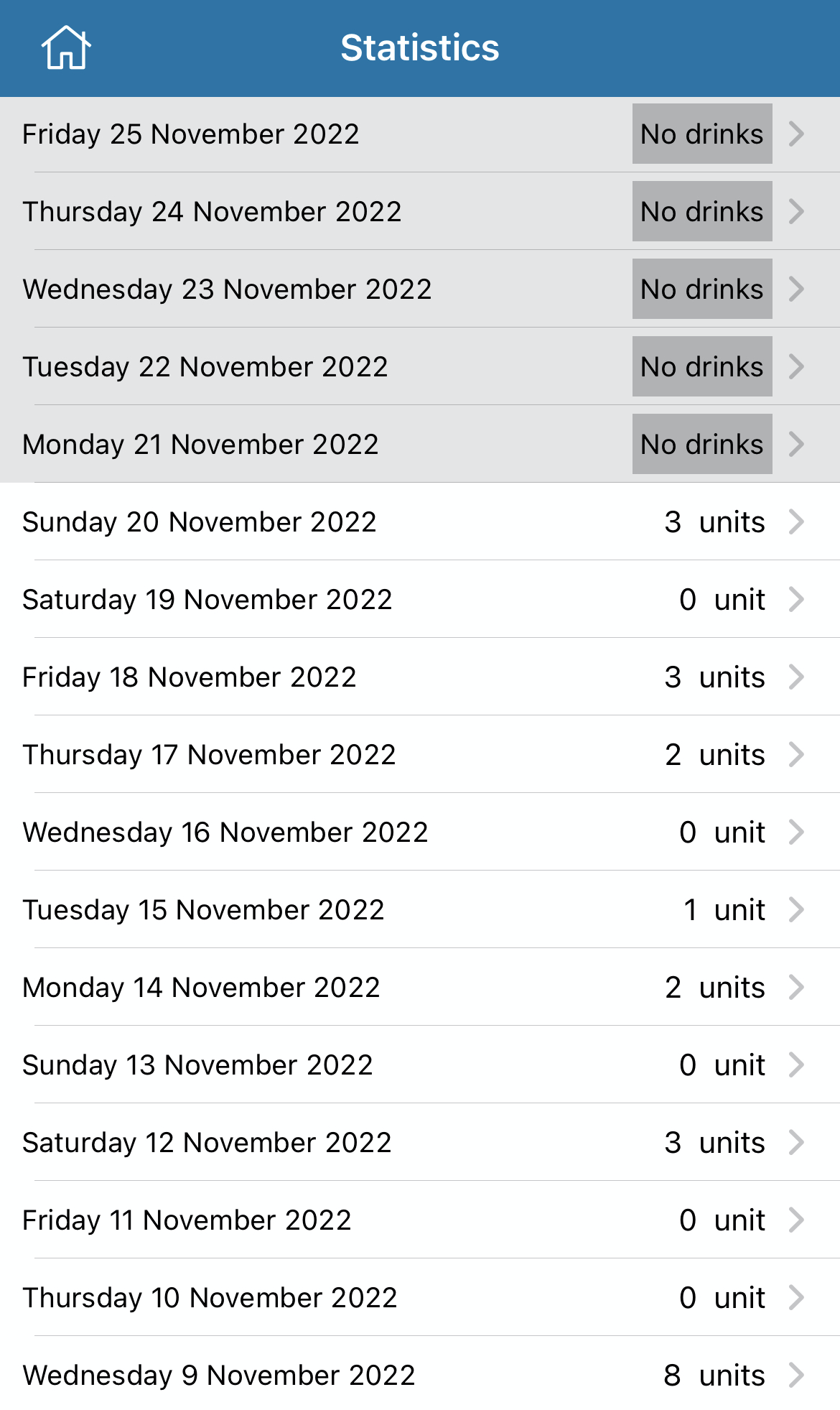** 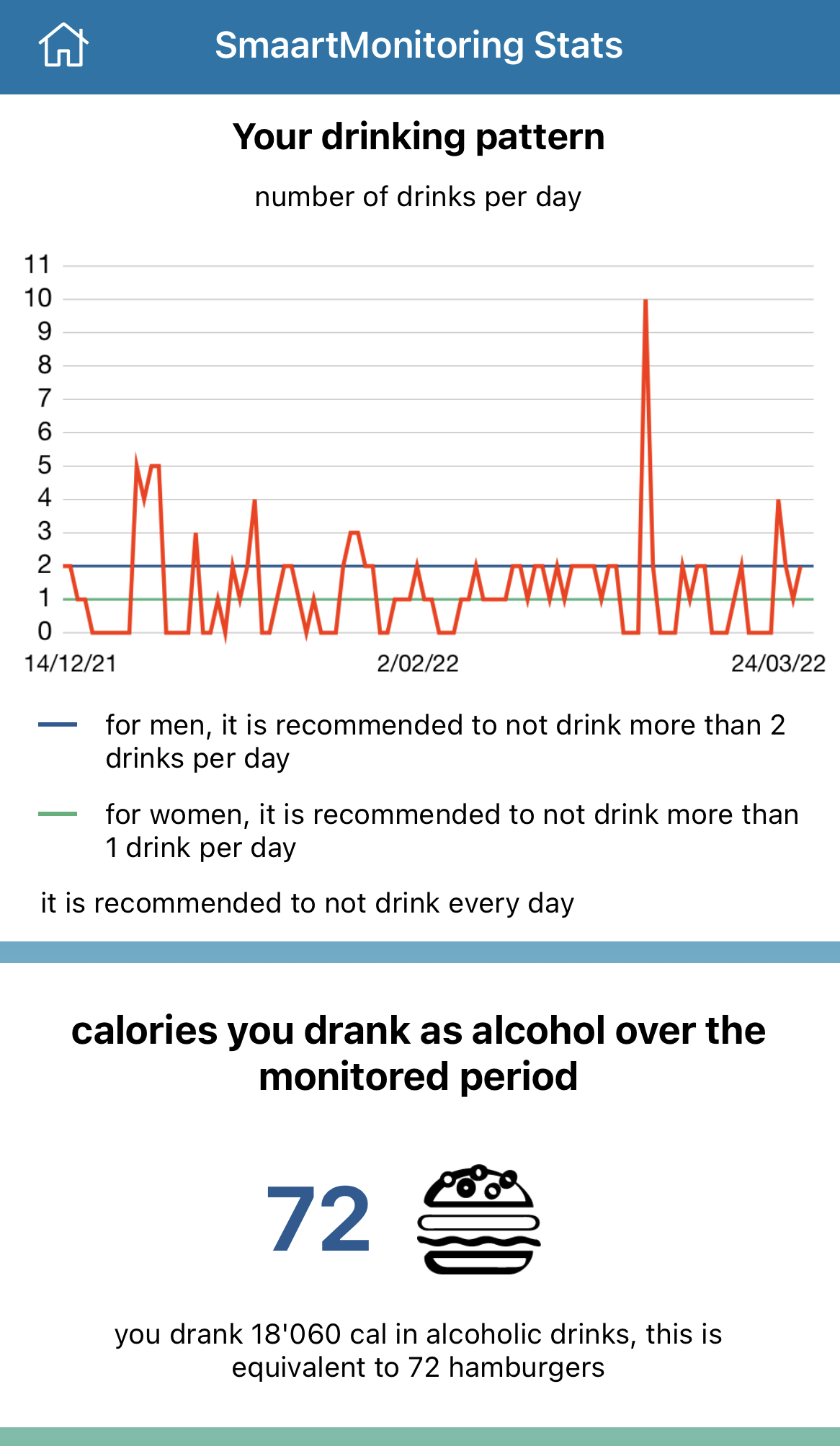  A shortcut was introduced for the “no drinks” option. Clicking on “no drinks” automatically records 0 drink. Otherwise, a screen with the various drinks options is presented for the user to choose from |

Figure 2 continued

| **Goal setting (Challenge)** | | |
| --- | --- | --- |
| **Pre-test 1 (prototype 1)** | **Pre-test 2 (prototype 2)** | **Final version** |
| 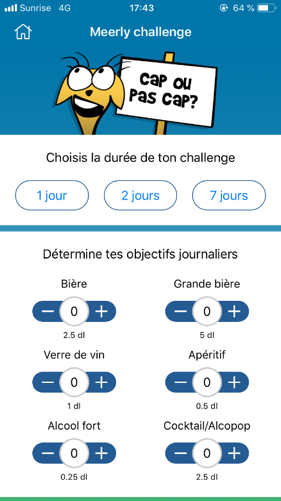 | **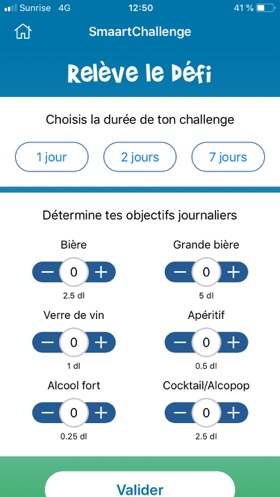** | 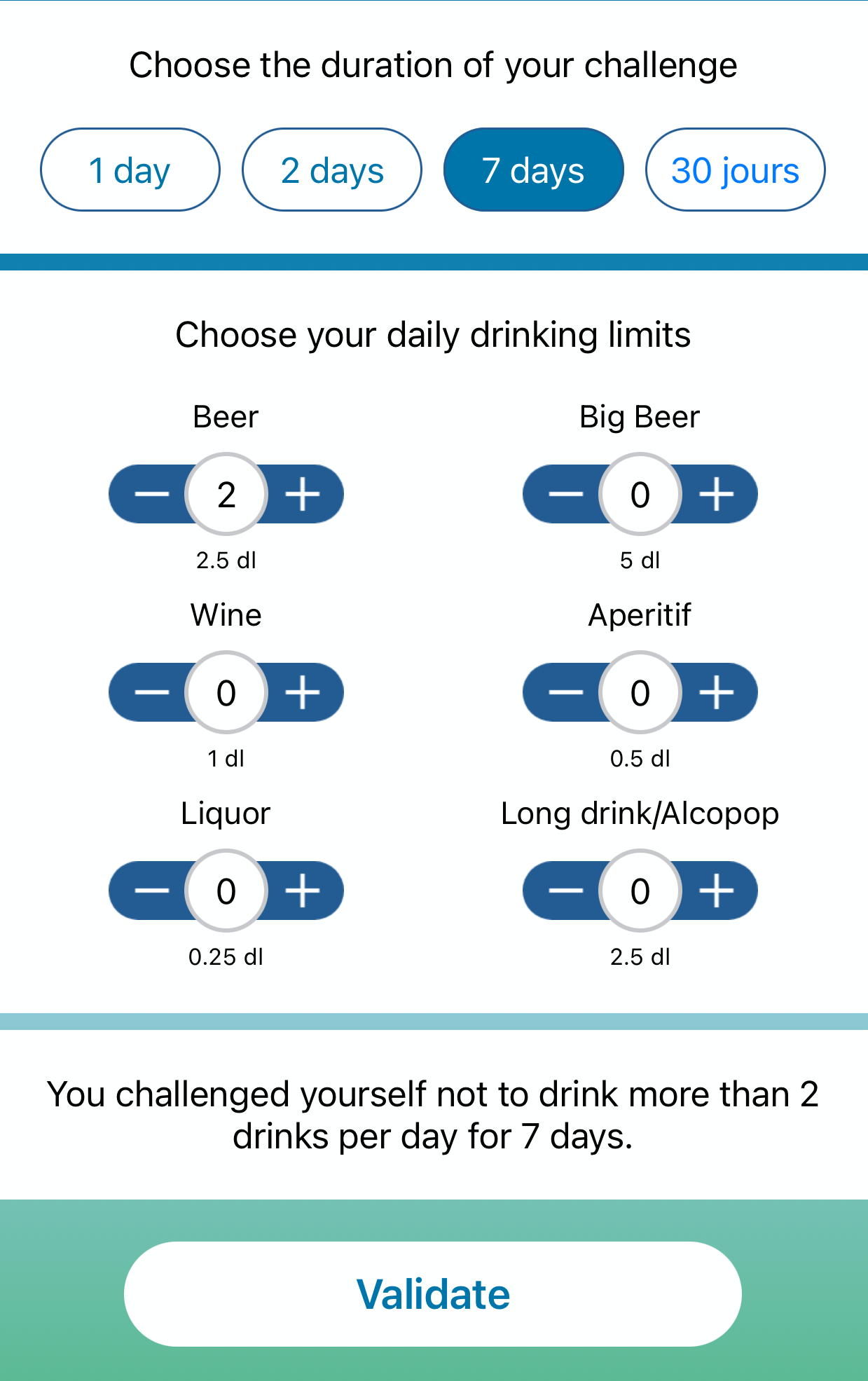 |
| 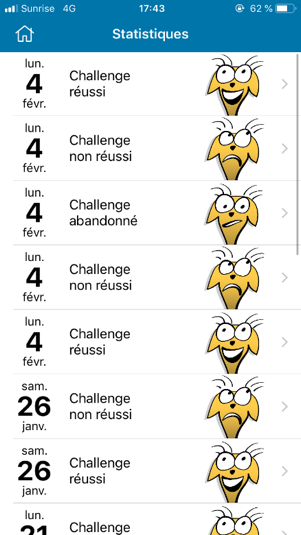 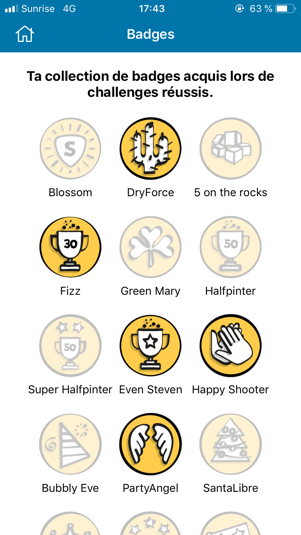 | 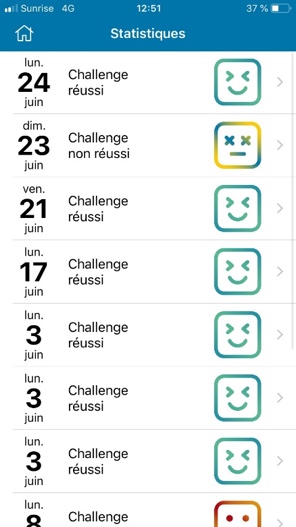 | 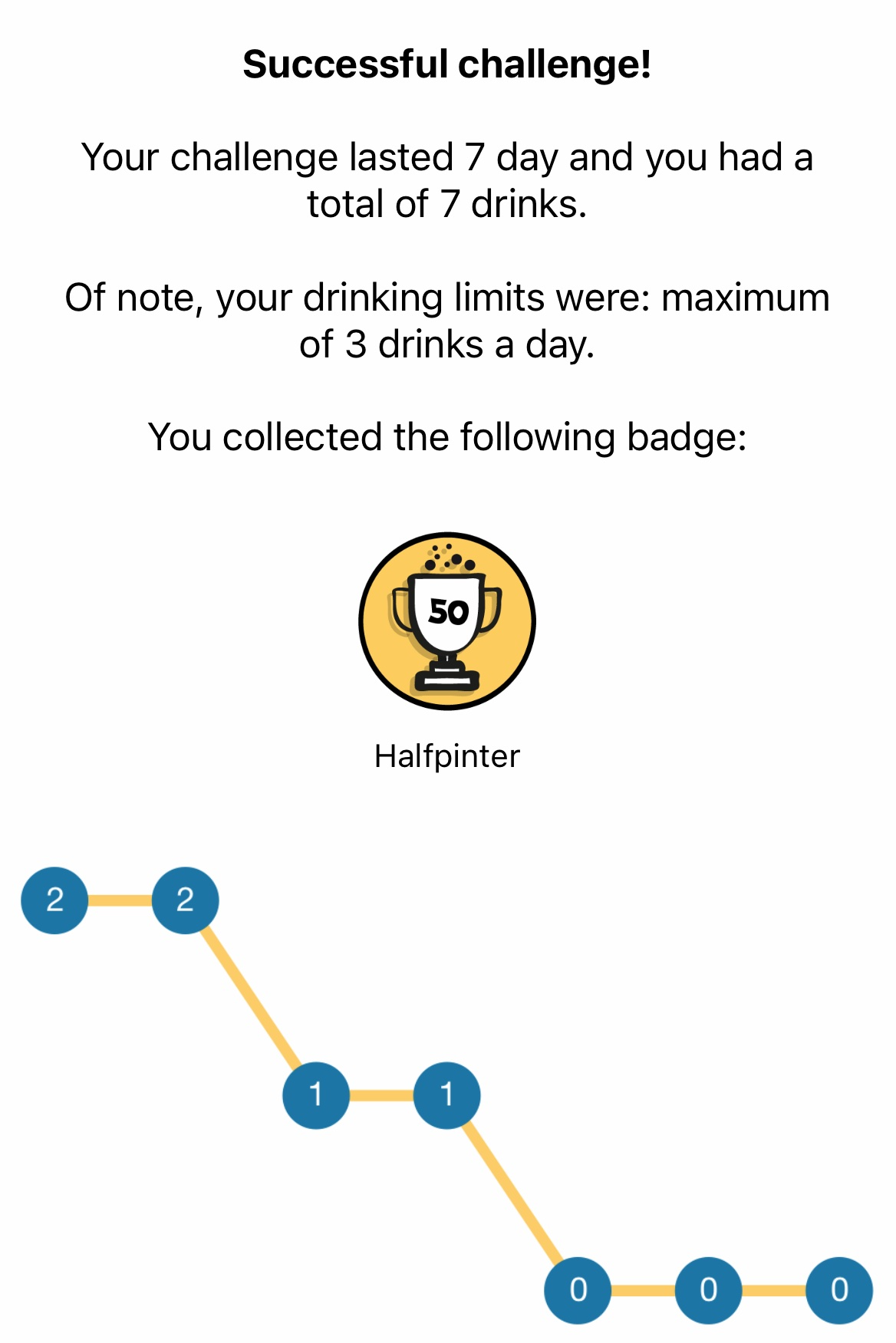 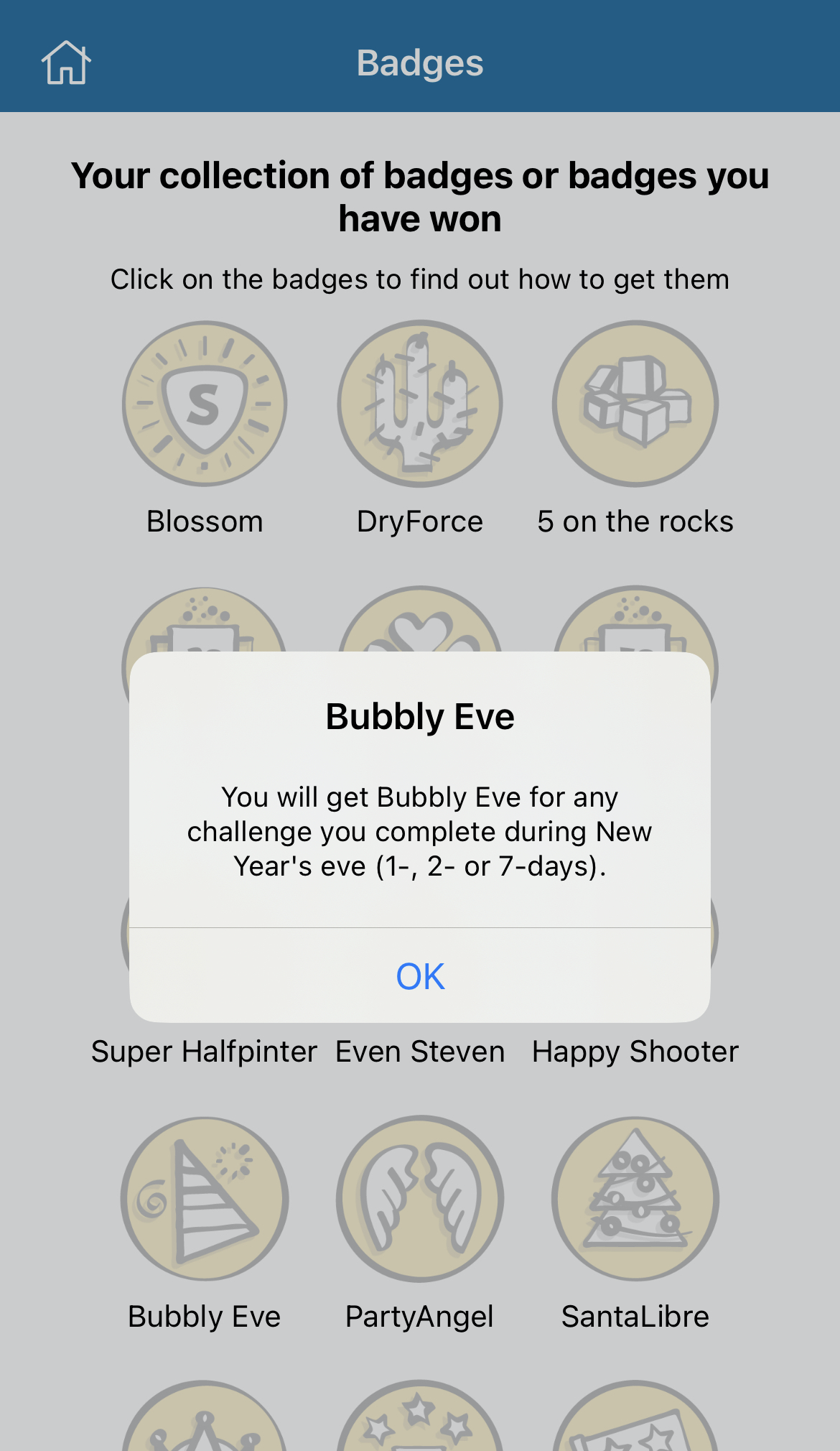  Badges: users can access information on how to earn a badge by clicking on it |

Figure 2 continued

| **Designated driver (Driver)** | | |
| --- | --- | --- |
| **Pre-test 1 (prototype 1)** | **Pre-test 2 (prototype 2)** | **Final version** |
| 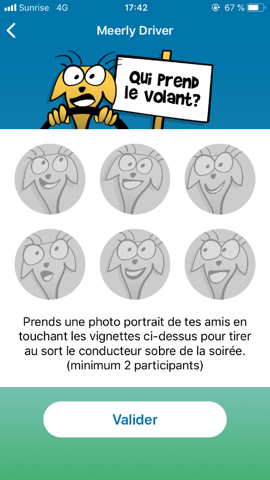 | **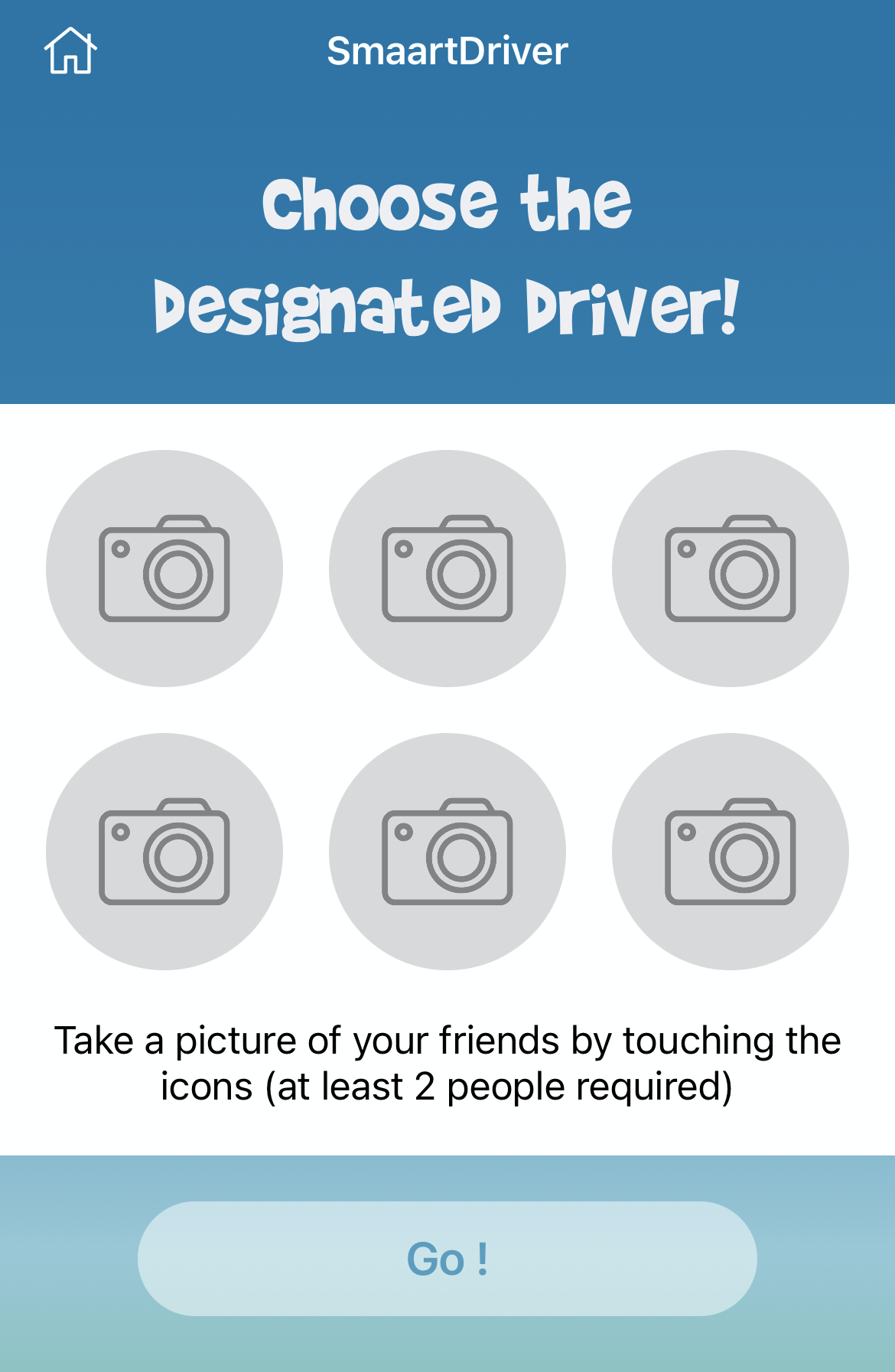** | |
| **Fact sheets (Pedia)** | | |
| **Pre-test 1 (prototype 1)** | **Pre-test 2 (prototype 2)** | **Final version** |
| 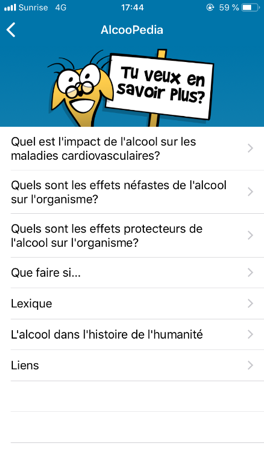 | 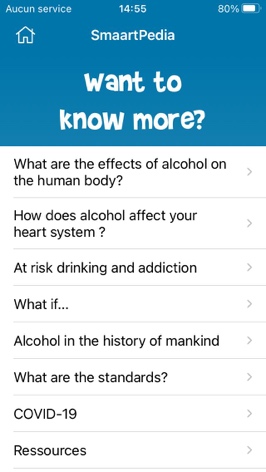 | |

Figure 2 end
