# Supplementary material for: Development of a Secondary Prevention Smartphone App for Students With Unhealthy Alcohol Use: Results From a Qualitative Assessment
Source: JMIR Hum Factors. 2023 Mar 7;10:e41088. doi: 10.2196/41088 (PMC10031438; doi:10.2196/41088)
Supplement: Multimedia Appendix 3 [file humanfactors_v10i1e41088_app3.docx]

| **INTERVIEW GRID**  Pre-test interview 2, group 1 (pre-test participants 1) |
| --- |

| **THEMES** | **QUESTIONS & REMINDERS** |
| --- | --- |
|  | |
| **General impressions**  **New version**  **Content of the application**  **Usefulness**  **Module not used** | What do you think of the new version of the application?  What made an impression on you? Why?  To what extent would you recommend the application to your friends, why?  Do you think your peers/friends would use the application (in its new version) regularly?   - Why?   What new things have you discovered with this new version of the app?  What new features did you like?  Which ones did you not like?  What do you think of the content of the application?  What did you find useful in the new version of the application?  What didn't you find useful in this application?  How much do you expect to continue using the application?   - Why?   *If one of the modules has not been used, bring print screens and evaluate with the person during the interview:*   - Why not use it? - What could be improved to make it more attractive to use? |
| **Ergonomics in general**  **Functioning**  **Module drop-out**  **Application design**  **Icon** | How was your experience with the application?  What do you think of the way the application works?  What difficulties have you encountered?  What do you think we could do to make the application easier to use?  *If there are dropouts (significant number) in any of the modules, bring print screens and evaluate with the person during the interview:*   - Why dropped-out?   What do you think of the design of the new version in general?  What do you think of the icon? |
| **Smaart Quizz**  **Smaart Test**  **Smaart Driver**  **Smaart Challenge**  **Smaart monitoring**  **Smaart Pedia** | What do you think of the Quizz module in the new version?  What do you think of the feedback?  What do you think of this module?  What do you think of the results?  What do you think?  What do you think of this module?  What do you think of the statistics (related to your challenges)  What do you think of this new module of the application?  How useful do you think this module is? Why or why not?  What do you think of the monitoring statistics?  How did it go using Smaart monitoring?  What do you think of the monitoring notification system?  There were no major changes on this module.  If you've looked at it again, what do you think? |
| **Notifications** | **Notifications**  What do you think about the content of the notifications?  How useful do you think notifications are?  What notifications could we add? |
|  | |
| **Any additional information** | What else you would like to share before we end this interview? |
