# Supplementary material for: Development of a Secondary Prevention Smartphone App for Students With Unhealthy Alcohol Use: Results From a Qualitative Assessment
Source: JMIR Hum Factors. 2023 Mar 7;10:e41088. doi: 10.2196/41088 (PMC10031438; doi:10.2196/41088)
Supplement: Multimedia Appendix 4 [file humanfactors_v10i1e41088_app4.docx]

| **INTERVIEW GRID**  Pre-test interview 2, group 2 (new participants) |
| --- |

| **THEMES** | **QUESTIONS & REMINDERS** |
| --- | --- |
|  | |
| **General impressions**  **Content of the application**  **Usefulness**  **Module not used** | What do you think of the application?   - What did you like most about this application? - What did you not like about this application?   What made an impression on you? Why?  To what extent would you recommend the application to your friends, why?  Do you think your peers/friends (define if necessary) would use this application regularly (in a non-study context, therefore not paid)?   - Why?   What do you think of the content of the application?  What did you find useful in this application?  What didn't you find useful in this application?  How much do you expect to continue using the application?   - Why?   *If one of the modules has not been used, bring print screens and evaluate with the person during the interview:*   - Why not use it? |
| **Ergonomics in general**  **Operation**  **Drop-out module**  **Application design** | How was your experience with the application?  What do you think of the way the application works?  What difficulties have you encountered?  What do you think we could do to make the application easier to use?  *If there are dropouts (significant number) in any of the modules, bring print screens and evaluate with the person during the interview:*   - Why abandoned?   What do you think of the design of the application in general?  What do you think of the icon? |
|  | |
| **Smaart Quizz**  **Smaart challenge**  **Smaart Test**  **Smaart Driver**  **Smaart Challenge**  **Smaart monitoring**  **Smaart Pedia** | What do you think of this module?  What was your reaction when you first read the feedback?  What do you think of the recommendations made at the bottom of the feedback?  According to the consumption indicated, the app proposes to make a challenge (*show example*).  What do you think?  What do you think of the badges (*show page if necessary*)?  What do you think of this module?  What do you think of the questions assessing alcohol consumption?  What do you think of this module?  What do you think of this module?  What do you think of the badges?  What do you think of this module?  How useful do you think this module is? Why or why not?  What do you think of the monitoring statistics?  How was the use of this module?  What do you think of this module?  Which pages did you find interesting and why?  What part(s) did you not like about this module and why?  What information do you think we should add/delete? |
| **Notifications**  **Activating/deactivating notifications** | What do you think about the content of the notifications?  How useful do you think notifications are?  What notifications could we add? |
| **Any additional information** | What else would you like to share before we end this interview? |
